# Analysis and modeling of Fano resonances using equivalent circuit elements

**DOI:** 10.1038/srep31884

**Published:** 2016-08-22

**Authors:** Bo Lv, Rujiang Li, Jiahui Fu, Qun Wu, Kuang Zhang, Wan Chen, Zhefei Wang, Ruyu Ma

**Affiliations:** 1 Microwave and Electromagnetic Laboratory, Harbin Institute of Technology, No.92, Xidazhi Street, Nangang District, Harbin City, Heilongjiang Province, China; 2 College of Information Science and Electronic Engineering, Zhejiang University, Hangzhou 310027, China

## Abstract

Fano resonance presents an asymmetric line shape formed by an interference of a continuum coupled with a discrete autoionized state. In this paper, we show several simple circuits for Fano resonances from the stable-input impedance mechanism, where the elements consisting of inductors and capacitors are formulated for various resonant modes, and the resistor represents the damping of the oscillators. By tuning the pole-zero of the input impedance, a simple circuit with only three passive components e.g. two inductors and one capacitor, can exhibit asymmetric resonance with arbitrary *Q*-factors flexiblely. Meanwhile, four passive components can exhibit various resonances including the Lorentz-like and reversely electromagnetically induced transparency (EIT) formations. Our work not only provides an intuitive understanding of Fano resonances, but also pave the way to realize Fano resonaces using simple circuit elements.

Fano resonance has received much attention due to the interesting physics such as distinctly asymmetric shape and high quality-factor (*Q*-factor)^1^. The interference of a discrete autoionized state with a continuum gives rise to characteristically asymmetric peaks in excitation spectra, which can be extended to the resonance scattering of quantum theory^2^,^3^,^4^. Recently, the classical oscillator systems enabled by plasmonic nanostructures and metamaterials have led to the achievement of asymmetric Fano-type transmission/reflection in the optical frequencies, which has open up a new perspective towards achieving high-precision nanoscale sensors^5^,^6^,^7^,^8^,^9^. Furthermore, the steep *Q*-factor profile promises applications in bio/chemical sensors^10^,^11^,^12^,^13^.

A discrete autoionized state and a continuum can be analogue of a broadband-bright mode and a narrowband-dark mode depending on the coupled approach with the incident light from free space^14^. The bright mode has a large scattering cross section and a low quality factor due to the radiation coupling, which is always excited directly by external energy. On the contrary, the dark mode normally has a significantly larger quality factor, which is only limited by the loss performance and excited indirectly by the bright mode^15^. The formation exhibits the interference phenomena, where constructive interference corresponds to resonant enhancement and destructive interference to resonant suppression of the transmission^16^,^17^. Furthermore, The circuit system which is an effective-mapping image of the classical mechanics can be devoted to the mechanism of Fano resonance^18^. In passive electric system, the inductance represents a behavior increasing with higher frequency in spectra domain, and the capacitance effects the opposite process. Accordingly, the electric resonance is the equilibrium state when the functionality of inductance and capacitance are balance. Based on this, the electric-dynamic equations of the ‘bright’ and ‘dark’ electric-resonant modes are established and imitate Fano resonance effectively. Nevertheless, the circuit structures of high-*Q*-factor resonances consist of numerous orders of electric resonances, and the solutions of dynamic-differential equations are extremely complicated. Therefore, the ultimate goal of simple structure and an effectively steady-convenient analysis of Fano-like resonance are highly desired.

In this paper, we formulate the series and parallel circuits consisting of inductors and capacitors for various-resonant modes, and the resistor represents the damping of the oscillators. Additionally, we propose the stable-input impedance mechanism of passive circuit system to mimic the functionality of the Fano resonance. Based on this theory, the pole-zero adjustment of the input impedance can implement arbitrary *Q*-factor asymmetric resonance flexiblely in simple circuit system which consists of only three passive components (such as two inductors and one capacitor). Furthermore, various resonances (such as Lorentz-like and reversely EIT formations) can be imitated by four electric components. This approach has well-defined Fano-like effective properties and opens up the possibilities to construct extremely high-*Q*-factor devices while maintaining the simplification of the system. Besides, they can be a guidance for design in microwave or optical circuits and, in particular, for periodic artificial electromagnetic materials. Additionally, it is interesting to note that the passive circuit approach and the theoretical propositions presented in this work processes to achieve high-precision and compressed-composition components^19^.

## Fano resonances without damping

**Figure 1:**
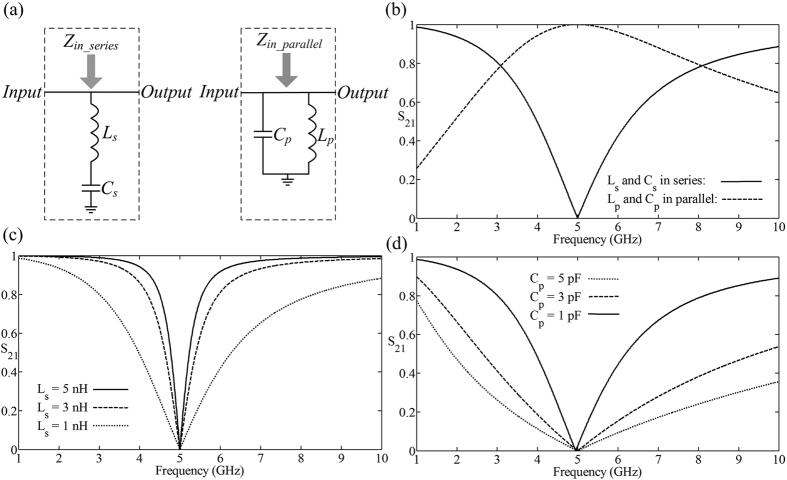
(**a**) The schematic of the series and parallel-*LC* circuit as the branch parallel in the main-energy thread. Here the inductors are *L*_*s*_ = *L*_*p*_ = *1.0132* *nH*, and the capacitors are *C*_*s*_ = *C*_*p*_ = *1* *pF*. (**b**) The transmittance S_21_ of the series and parallel-*LC* circuit system, for the series-circuit branch parallel in system, the reflected-resonant frequency 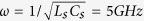, and the parallel-circuit branch parallel in system, the transparent-resonant frequency 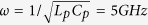. (**c**) The transmittance of series circuit with various *L*_*s*_ = 5 *nH*, 3 *nH*, 1 *nH* and the corresponding capacitor *C*_*s*_ = 0.2026 *pF*, 0.3377 *pF*, 1.1032 *pF* containing the resonant frequency *ω*_*s*0_ = 5 *GHz*. (**d**) The transmittance of parallel circuit with various *C*_*p*_ = 5 *pF*, 3 *pF*, 1 *pF* and the corresponding inductor *L*_*p*_= 0.2026 *nH*, 0.3377 *nH*, 1.1032 *nH* containing the resonant frequency *ω*_*p*0_ = 5 *GHz*.

First, we consider Fano resonances without damping, where the circuits are schematically shown in Fig. 1(a). We calculate the stable-input impedance of the circuit which is embedded in the single-input-single-output (SISO) system^20^, and tune its pole-zero^21^. The transmittance is defined as *S*_21_ = *P*_*output*_/*P*_*input*_, where *P*_*input*_ and *P*_*output*_ are the incident and transmitted power, respectively. The stable-input impedance of series inductor-capacitor (*LC*) circuit and parallel *LC* circuit are given as


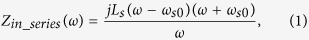


and


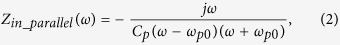
respectively, where the resonant frequencies 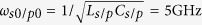 depend on the inductor *L*_*s/p*_ = 1.0132 *nH* and the capactor *C*_*s/p*_ = 1 *pF*. In Eq. (1), the stable-input impedance of series *LC* circuit *Z*_*in_series*_ has the zeros *ω* = ±*ω*_*s*0_ and the poles *ω* = 0. Here, the negative frequency *ω* = −*ω*_*s*0_ are ignored due to its physical-meaningless. Then, the input impedance of series circuit is shorted to the ground at the zero *ω* = *ω*_*s*0_, which leads the input energy total reflected and the transmittance is lowest *S*_21_ = 0 as the solid line in Fig. 1(b). In Eq. (2), the input-impedance function of parallel *LC* circuit has the zero *ω* = 0, and the poles *ω* = ±*ω*_*p*0_. Excluding the physical-meaningless pole *ω* = −*ω*_*p*0_, the input-impedance of parallel *LC* circuit is infinite at the pole point *ω* = *ω*_*p*0_ and the transmittance is all-pass *S*_21_ = 1 as the dashed line in Fig. 1(b). From Eq. (1, 2), the steep in the vicinity of *ω*_*s*0_ and *ω*_*p*0_ is proportional to the inductor *L*_*s*_ in the series circuit, and inversely proportional to the capacitor *C*_*p*_ in the LC-parallel circuit. Therefore, the Q factor can be adjusted by the inductor *L*_*s*_ and the capacitor *C*_*p*_ as shown in Fig. 1(c,d), meanwhile, the corresponding capacitor *C*_*s*_ and inductor *L*_*p*_ is modified for the remaining of resonant frequency *ω*_0*s*/*p*_ = 5 GHz.

Here the *Q*-factor is expressed as *Q* = *ω*_0_/(*ω*_*H*_ − *ω*_*L*_), where *ω*_0_ is the central resonant frequency, and *ω*_*L*_, *ω*_*H*_ are the half-amplitude frequencies lower and higher than *ω*_0_. In Fig. 1(c), the series-*LC Q*-factor are 10.8, 6.5 and 2 for the various inductor *L*_*s*_ = 5 *nH*, 3 *nH* and 1 *nH*. which presents the series-*LC* resonance sharper with decreasing series inductor *L*_*s*_. In Fig. 1(d), the parallel-LC *Q*-factor are 2.27, 1.36 and 0.45 for the various capacitor *C*_*p*_ = 5 *pF*, 3 *pF* and 1 *pF*, which presents the parallel-*LC* resonance sharper with increasing parallel capacitor *C*_*p*_.

**Figure 2:**
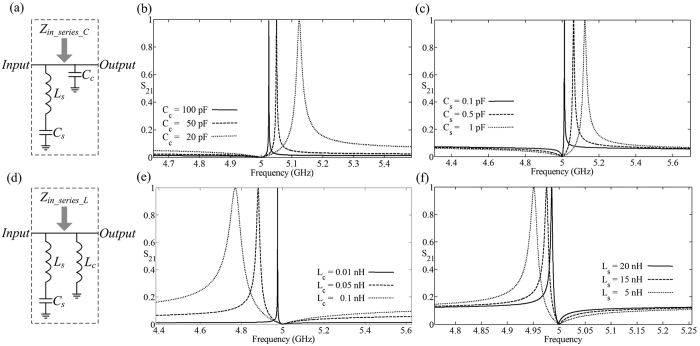
(**a**) The schematic of the complementary capacitor *C*_*c*_ parallel in the series LC circuit. (**b**) The transmittance of the circuit system in Fig. 2(a) with different complementary capacitor *C*_*c*_ = *20* *pF, 50* *pF* and *100* *pF*. (**c**) The transmittance of the circuit system in Fig. 2(a) with different series capacitor *C*_*s*_ = *0.1* *pF, 0.5* *pF* and *1* *pF*. (**d**) The schematic of the complementary inductor *L*_*c*_ parallel in the series *LC* circuit. (**e**) The transmittance of the circuit system in Fig. 2(d) with different complementary inductor *L*_*c*_ = *0.1* *nH, 0.05* *nH* and *0.01* *nH*. (**f**) The transmittance of the circuit system in Fig. 2(d) with different series inductor *L*_*s*_ = *5* *nH, 15* *nH* and *20* *nH*.

Based on the above analysis, we can build the Fano-like asymmetric resonance by a series-*LC* circuit which represents the narrowband-dark mode coupling with a capacitor or an inductor as the broadband-bright mode, as shown in Fig. 2(a,d). Here we use the stable-input impedance method instead of oscillators-dynamic equations in spectra domain to reveal the mechanism of the asymmetric-coupling modes. In Fig. 2(a), the complementary capacitor *C*_*c*_ is added parallel to the series-LC resonance, and the the stable-input impedance of this circuit system is:


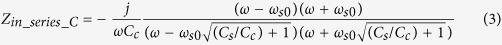


Abandoning the physical meaningless solutions, we get the pole of stable-input impedance 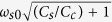 in Eq. (3) which is greater than the zero *ω*_*s*0_. In addition, the zeros and poles are corresponding to the reflect and transparent resonant frequencies respectively in main-energy thread. Therefore, the transmittance can steep down to zero *ω*_*s*0_ at the higher-frequent pole 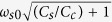 with the coefficient 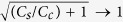, and presents the formation of Fano-like asymmetric resonance and and high-Q factor. Further, we can get the infinite-*Q*-factor by turning the pole greatly close to the zero through increasing the complementary capacitor *C*_*c*_ and decreasing the series capacitor *C*_*s*_. Here we maintain the series-resonant frequency *ω*_*s*0_ = 5 *GHz*, and increase the complementary capacitor *C*_*c*_ = 20 *pF*, 50 *pF*, 100 *pF*, that leads to the transparent resonance 5.132 *GHz*, 5.050 *GHz* and *5.025* *GHz* closing to the reflect resonance *ω*_*s*0_ = 5 *GHz* gradually, and the resonance becomes sharper, as shown in Fig. 2(b). When the complementary capacitor *C*_*c*_ = 20 *pF* and decreasing the series capacitor *C*_*s*_ = 1 *pF*, 0.5 *pF*, 0.1 *pF*, under the conditions of the series inductor *L*_*s*_ changing correspondingly for maintaining the series-resonant frequency *ω*_*s*0_ = 5 *GHz*, the transparent resonance is 5.132 *GHz*, 5.062 *GHz*, 5.013 *GHz* closing to the reflect resonance *ω*_*s*0_ = 5 *GHz* gradually, and the *Q*-factor becomes higher, as shown in Fig. 2(c).

The complementary inductor *L*_*c*_ is parallel-added in the series-*LC* circuit, as shown in Fig. 2(d), and the the stable-input impedance is:


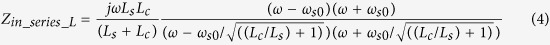


Abandoning the physical meaningless solutions, we get the pole 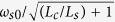 in Eq. (4) lower than the zero *ω*_*s*0_. Therefore, the transmittance can steep down to zero at the pole 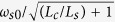 located lower than the zero *ω*_*s*0_ when the coefficient 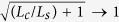, and presents the formation of Fano-like asymmetric resonance. Further, we can get the infinite-*Q*-factor by turning the pole point close to the zero point through decreasing the complementary inductor *L*_*c*_ and decreasing the series inductor *L*_*s*_. Here we maintain the series-resonant frequency *ω*_*s*0_ = 5 *GHz*, and decrease the complementary inductor *L*_*c*_ = 0.1 *nH*, 0.05 *nH*, 0.01 *nH*, that leads the transparent resonance 4.770 *GHz*, 4.881 *GHz*, 4.976 *GHz* closes to the reflect resonance *ω*_*s*0_ = 5 *GHz* gradually, and the *Q*-factor becomes higher, as shown in Fig. 2(e). When the complementary capacitor *L*_*c*_ = 0.1 *nH* is constant and increasing the series inductor *L*_*s*_ = 5 *nH*, 15 *nH*, 20 *nH*, under the conditions of the series inductor changing correspondingly for maintaining the series-resonant frequency *ω*_*s*0_ = 5 *GHz*, the transparent resonance is 4.951 *GHz*, 4.976 *GHz*, 4.986 *GHz* closing to the reflect resonance *ω*_*s*0_ = 5 *GHz* gradually, and the *Q*-factor becomes higher, as shown in Fig. 2(f).

In Fig. 2(b), the *Q*-factor is 2513, 1263 and 197 for *C*_*c*_ = 100 *pF*, 50 *pF* and *20* *pF*, which presents the resonance sharper with the increasing the complementary capactor *C*_*c*_. In Fig. 2(c), the *Q*-factor is 1671, 361.6 and 197.1 for *C*_*s*_ = 0.1 *pF*, 0.5 *pF* and *1* *pF*, which presents the resonance sharper with decreasing the series capacitor *C*_*s*_. In Fig. 2(e), the *Q*-factor is 4976, 203.4 and 51.85 for *L*_*c*_ = 0.01 *nH*, 0.05 *nH* and *0.1* *nH*, which presents the resonance sharper with decreasing the complementary inductor *L*_*c*_. In Fig. 2(f), the *Q*-factor is 831, 712 and 225 for *L*_*s*_ = 20 *nH*, 15 *nH* and *5* *nH*, which presents the resonance sharper with increasing the complementary inductor *L*_*s*_.

**Figure 3:**
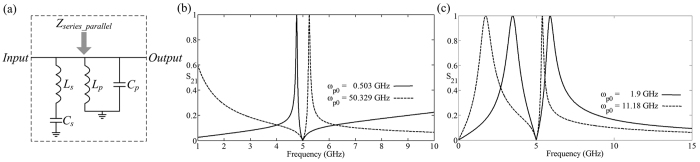
(**a**) The schematic of the series and parallel resonant circuits are parallel in the main-energy thread. (**b**) The transmittance of the circuit system in Fig. 3(a) with different parallel-resonant frequencies *ω*_*p*0_ = 0.503 *GHz*, 50.329 *GHz*. (**c**) The transmittance of the circuit system in Fig. 3(a) with different parallel-resonant frequencies *ω*_*p*0_ = 1.9 *GHz*, 11.18 *GHz*.

We build the series and parallel resonant circuits parallel in the main-energy thread as shown in Fig. 3(a), and analyze the stable-input impedance:


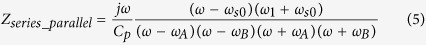
where the poles expressed as 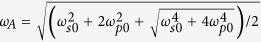 and 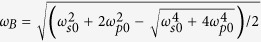. Here we maintain the series-circuit elements *L*_*s*_ = 1.0132 *nH, C*_*s*_ =1 *pF* and thus the series-resonant frequency *ω*_*s*0_ = 5 *GHz*. Abandoning the physical meaningless solutions, when the series and parallel resonant frequencies satisfying *ω*_*p*0_ _≪_ *ω*_*s*0_, the pole *ω*_*B*_ of Eq. (5) satisfies *ω*_*B*_ ≈ 0, and the other pole *ω*_*A*_ is little higher than the zero *ω*_*s*0_. Therefore, the closing of pole and zero can construct transparent-asymmetric and high-*Q*-factor resonance. Based on the above analysis, we can set the parallel elements *L*_*p*_ = 1.1032 *nH, C*_*p*_ =0.1 *pF*and thus the parallel-resonant frequency *ω*_*p*0_ = 0.503 *GHz*. which leads to the transparent resonant frequency *ω*_*B*_ = 5.246 *GHz* closing to the zero *ω*_*s*0_, shown as the dashed line in Fig. 3(b). When the series and parallel resonant frequencies satisfying *ω*_*p*0_ _≫_ *ω*_*s*0_, the pole 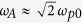 is far from *ω*_*s*0_, and the other pole *ω*_*B*_ ≈ *ω*_*s*0_ is little lower than the zero *ω*_*s*0_ in Eq. (5) which constructs the transparent-asymmetric and high-Q-factor resonance. We set the parallel elements *L*_*p*_ = 0.1 *nH, C*_*p*_ = 0.1 *pF* and thus the parallel-resonant frequency *ω*_*p*0_ = 50.329 *GHz*, which leads to the transparent resonant frequency *ω*_*B*_ = 4.768 *GHz* closing to the zero *ω*_*s*0_, shown as the solid line in Fig. 3(b). When we set the parallel elements *L*_*p*_ = 1.0132 *nH, C*_*p*_ = 7 *pF*, and parallel-resonant frequency *ω*_*p*0_ = 1.9 *GHz*. Thus, from the solution of Eq. (5), the pole *ω*_*B*_ = 4.753 *GHz* is located lower than the zero *ω*_*s*0_ which forms the Lorentz-like resonance, and the other pole *ω*_*A*_ = 6.392 *GHz* is little higher than the zero *ω*_*s*0_ which forms the transparent-asymmetric and high-*Q*-factor resonance, shown as the dashed line in Fig. 3(c). When the poles *ω*_*s*0_ − *ω*_*B*_ = *ω*_*A*_ − *ω*_*s*0_ distribute even around the zero *ω*_*s*0_, the two resonant frequencies locate asymmetric and a sharp reflect-resonance is formed at the zero *ω*_*s*0_ which is likely a converse reversely EIT formation. Here we set the parallel elements *L*_*p*_ = 0.2993 *nH*and *C*_*p*_ = 5 *pF*, thus, the the parallel resonant frequency *ω*_*p*0_ = 4.1144 *GHz*. The transparent resonant frequencies *ω*_*A*_ = 5.895 *GHz, ω*_*B*_ = 3.49 *GHz*, which forms two mirror symmetrical resonance, as shown the solid line in Fig. 3(c), and the reflect resonance *ω*_*s*0_ = 5 *GHz* is also like a reversely EIT phenomenon.

## The asymmetric resonance with damping

**Figure 4:**
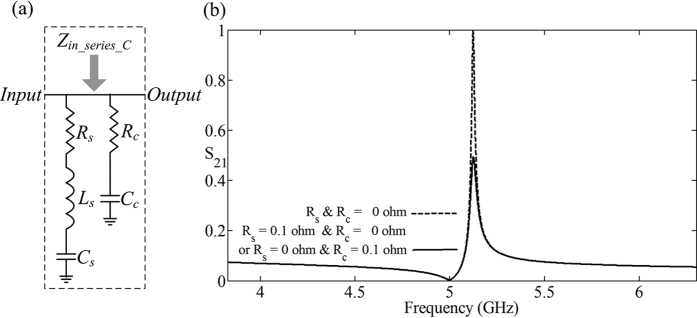
The schematic of the complementary capacitor *C*_*c*_ = 20 *pF* parallel in the series LC circuit consisting of the series inductor *L*_*s*_ = 1.0132 *nH* and the series capacitor *C*_*s*_ = 1 *pF* with damping which is represented by the resistors *R*_*s*_ and *R*_*c*_. (**b**) The transmittance of the circuit system in Fig. 4(a) with different resistors *R*_*s*_ = 0.1 *ohm* and *R*_*c*_ = 0.1 *ohm*.

Here we add the resistors *R*_*s*_, *R*_*c*_ as damping in the series resonant circuit consisting of the series inductor *L*_*s*_ = 1.0132 *nH*, the series capacitor *C*_*s*_ = 1 *pF* and the complementary capacitor *C*_*s*_ = 20 *pF*, as shown in Fig. 4(a). We set the resistor *R*_*s*_ = 0.1 *ohm* and *R*_*c*_ = 0.1 *ohm* respectively, which leads to the amplitude of the transmittance is lower than no-damping, but the shape of the asymmetric resonance remains unchanged, as shown in Fig. 4(b).

## Conclusion

In conclusion, we show that the Fano resonance can be interpreted as an analogy with the stable-input impedance mechanism by taking passive circuit system as an example. Based on the circuit theory, only three passive components (such as two inductors and one capacitor) can mimic arbitrary *Q*-factor asymmetric resonance flexiblely by adjusting the pole-zero of the impedance. Furthermore, four passive components can imitate the various resonance (such as Lorentz-like and reversely EIT formations). Besides, our work provides an intuitive understanding of the Fano resonance using briefly electric formation and processes to achieve high-precision by compressed-composition components.

## Additional Information

**How to cite this article**: Lv, B. *et al*. Analysis and modeling of Fano resonances using equivalent circuit elements. *Sci. Rep.* **6**, 31884; doi: 10.1038/srep31884 (2016).
